# The associations among serum vitamin D concentration, systemic immune-inflammation index, and lifestyle factors in Chinese adults: a cross-sectional analysis

**DOI:** 10.3389/fnut.2025.1543925

**Published:** 2025-05-30

**Authors:** Feiin Chan, Chongsong Cui, You Peng, Zhenjie Liu

**Affiliations:** ^1^School of Second Clinical Medicine, Guangzhou University of Chinese Medicine, Guangzhou, China; ^2^The Second Affiliated Hospital of Guangzhou University of Chinese Medicine, Guangzhou, China; ^3^Guangdong Provincial Hospital of Chinese Medicine, Guangzhou, China; ^4^State Key Laboratory of Chinese Medicine Syndrome, Guangzhou, China

**Keywords:** vitamin D, 25-hydroxyvitamin D, 25(OH)D, systemic chronic inflammation, inflammation, systemic immune-inflammatory index, SII, lifestyle

## Abstract

**Introduction:**

Vitamin D is a crucial lipid-soluble hormone that has been demonstrated to be closely associated with systemic chronic inflammation and various diseases, including metabolic disorders, cardiovascular conditions, autoimmune diseases, cancer, and aging. The pathological underpinnings of these diseases are intricately linked to systemic chronic inflammation. The systemic immune-inflammation index (SII), an emerging biomarker, offers a more comprehensive reflection of the state of systemic inflammation and the immune response by integrating interactions among diverse immune cell types. This study aimed to evaluate the association between serum 25-hydroxyvitamin D (25(OH)D) concentrations and the SII while further exploring the association between vitamin D levels and systemic chronic inflammation in the Chinese population. Additionally, it analyses how lifestyle choices and dietary habits influence vitamin D levels.

**Methodology:**

This investigation employed a cross-sectional research design involving 1,177 participants aged 18–90 years who were selected from Zhong Hospital in Guangdong Province, China, through screening procedures. Ultimately, data from 726 participants were analysed following the screening and inclusion criteria. The participants were categorized into three groups on the basis of their serum vitamin D concentrations: the deficient group (SVD < 20 ng/mL), suboptimal group (SVD ≥ 20 but<30 ng/mL), and optimal group (SVD ≥ 30 ng/mL). Physiological indicators, medical history, lifestyle and dietary habits were collected; the SII was calculated via the following formulas: SII=platelet count×neutrophil count/lymphocyte count . Statistical comparisons of the intergroup differences were subsequently conducted, followed by logistic regression and correlation analyses. Subsequently, intergroup differences were assessed, and logistic regression and correlation analysis were performed.

**Results:**

The findings indicated that the SII value in the vitamin D-deficient group was significantly higher than that in the optimal group (*p* < 0.05). Furthermore, individuals in this deficient category presented elevated levels of metabolic markers such as total cholesterol, low-density lipoprotein cholesterol (LDL-C), fasting blood glucose, glycated hemoglobin A1c (HbA1c), and uric acid alongside unhealthy lifestyle practices characterized by frequent consumption of cold foods and sugary beverages coupled with high work-related stressors and prolonged air conditioning use (*p* < 0.05). Conversely, high-density lipoprotein cholesterol (HDL-C) was positively correlated with vitamin D status (*p* < 0.05).

**Conclusion:**

These results substantiate the association between vitamin D levels and the SII while suggesting that interventions targeting lifestyle modifications may positively impact vitamin D status, thereby ameliorating systemic chronic inflammation. Although this study provides preliminary evidence regarding the interplay between vitamin D deficiency and systemic inflammatory processes, further investigations are warranted to elucidate the underlying biological mechanisms and explore potential strategies for regulating vitamin D concentrations through improved lifestyles and dietary choices.

## Introduction

Vitamin D, as a critical lipid-soluble steroid hormone, plays an essential role in maintaining calcium and phosphorus metabolism and immune homeostasis in the human body. Recent research, particularly during the COVID-19 pandemic, has further highlighted vitamin D’s pivotal role in regulating immune function and influencing disease susceptibility (1).

The biosynthesis and metabolism of vitamin D involve a complex physiological process. In the skin, 7-dehydrocholesterol (7-DHC) is converted into vitamin D3 under ultraviolet B (UVB) radiation. Vitamin D3 is then catalyzed by 25-hydroxylase (CYP2R1) in the liver to form 25-hydroxyvitamin D3 [25(OH)D3], which serves as the optimal biomarker for assessing vitamin D nutritional status and was used as the detection index in this study. Subsequently, 25(OH)D3 is converted into its biologically active form, 1,25-dihydroxyvitamin D3 [1,25(OH)2D3], through the action of 1α-hydroxylase (CYP27B1) in the kidneys. Besides endogenous synthesis, dietary sources such as fatty fish, cod liver oil, egg yolks, mushrooms, and fortified foods also contribute significantly to vitamin D intake. The biological activity of 1,25(OH)2D3 is mediated by binding to vitamin D receptors (VDR) in the cell nucleus, thereby regulating the expression of multiple target genes involved in various physiological functions (1, 2).

Increasing evidence suggests that vitamin D is intricately linked with systemic chronic inflammation and is correlated with various diseases, such as metabolic disorders, cardiovascular conditions (3–10). The underlying pathology of these conditions is closely associated with systemic chronic inflammation (11–19). However, current research findings regarding the association between vitamin D and systemic inflammatory states lack comprehensiveness (1).

Emerging evidence suggests that vitamin D plays a crucial role in modulating systemic chronic low-grade inflammation. Specifically, 25(OH) D exerts anti-inflammatory effects via multiple mechanisms, including inhibiting the nuclear factor κβ (NF-κβ) signaling pathway, regulating T-cell subset differentiation, and downregulating the production of pro-inflammatory cytokines such as IL-6 and TNF-*α* (20–22). However, due to variations in study populations and the inherent limitations of single biomarkers in reflecting complex inflammatory states, existing studies on the correlation between vitamin D and traditional inflammatory markers (e.g., C-reactive protein, IL-6) have yielded heterogeneous conclusions. The systemic immune-inflammation index (SII), a novel composite inflammatory indicator calculated as SII = platelet count ×neutrophil count/lymphocyte count, provides a more comprehensive reflection of systemic inflammation and immune balance. For example, a large cohort study from the UK Biobank demonstrated that after adjusting for body weight, vitamin D deficiency was significantly associated with elevated SII values, whereas no significant association was observed with CRP levels (23). This finding suggests that SII may be more sensitive to metabolic-related chronic low-grade inflammation than acute inflammatory responses. Additionally, in patients with ischemic heart disease, the association between SII and vitamin D levels was stronger in the acute coronary syndrome (ACS) subgroup (24), indicating SII’s potential utility in assessing immune-inflammatory imbalances and chronic disease risks. Compared to traditional inflammatory markers, SII exhibits higher sensitivity in evaluating chronic metabolic inflammation and predicting cardiovascular disease risk (2, 16, 18, 25, 26), likely due to its ability to directly reflect immune cell-mediated vascular endothelial injury and plaque instability. The uniqueness of SII lies in its integration of dynamic changes across different immune cell subsets: platelets represent coagulation-inflammation interactions, neutrophils indicate innate immune activation, and lymphocytes reflect adaptive immune regulation. This multidimensional characteristic enables SII to capture the broad regulatory effects of vitamin D on the immune system, such as NF-κβ pathway inhibition and Treg/Th17 balance modulation, which single indicators like CRP cannot fully encompass. Despite these insights, the mechanistic basis underlying the association between SII and vitamin D remains underexplored and warrants further investigation in conjunction with vitamin D’s immune-modulatory functions. Notably, epidemiological studies examining the association between vitamin D status and SII are limited, particularly in Chinese populations where relevant evidence is scarce.

Vitamin D deficiency and insufficiency are highly prevalent among Chinese adults. A study reports that 17.9% of middle-aged adults exhibit vitamin D deficiency, while 42.9% show insufficiency (27). Another study reveals that among elderly individuals aged 50–70 years, the prevalence rates for deficiency and insufficiency are 69.2 and 24.4%, respectively (28). The vitamin D nutritional status of the Chinese population is influenced by a combination of environmental, behavioral, and genetic factors. First, the studied southern region exhibits high urbanization, where atmospheric pollutants (e.g., PM2.5) significantly scatter and absorb UVB radiation. Combined with the predominantly indoor lifestyle of the local population, these factors restrict endogenous vitamin D synthesis in the skin. Second, cultural preferences for aesthetic appearances lead to widespread use of sun protection products (SPF ≥ 30) and physical sunblock measures, further reducing UVB exposure. Moreover, the generally low intake of dairy products among Chinese individuals, coupled with a high prevalence of lactose intolerance (29), contributes to insufficient dietary vitamin D intake. Other modern lifestyle characteristics, such as psychological stress, smoking, alcohol consumption, and dietary habits favoring raw or cold foods, may also influence vitamin D status through various pathways. Therefore, systematically investigating the associations between vitamin D status and lifestyle factors holds significant implications for public health.

Based on the available evidence, we propose the following scientific hypotheses: (1) Serum 25(OH) D levels exhibit a negative correlation with SII values, suggesting that vitamin D insufficiency may promote chronic low-grade inflammation and increase the risk of metabolic diseases; (2) Healthy lifestyle behaviors contribute to maintaining adequate vitamin D levels, thereby exerting protective effects against chronic inflammation and related diseases. This study aims to evaluate lifestyle factors from multiple dimensions to provide new evidence-based medical insights for preventing vitamin D deficiency and facilitating early intervention of chronic inflammation-related diseases in the Chinese population, which is of great value for public health practice.

## Materials and methods

### Study design and population

This investigation employed a cross-sectional design, utilizing data from a national key research and development initiative (2018YFC2002503) conducted at the Guangdong Provincial Hospital of Traditional Chinese Medicine. The study aimed to demonstrate the early identification, intervention, and comprehensive service technology for metabolic syndrome within the framework of traditional Chinese medicine. Ethical approval from the Ethics Committee of Guangdong Traditional Chinese Medicine Hospital (approval number: BE2021-156-01) and was registered with the Chinese Clinical Trial Registry (registration number: ChiCTR2100054654). All participants provided written informed consent, and the study adhered to the principles of the Declaration of Helsinki.

Data collection occurred between August 2020 and January 2022 and included individuals admitted to inpatient services or outpatient clinics or who were undergoing health examinations at the hospital, resulting in a total of 1,177 cases.

The inclusion criteria were as follows: (1) aged between 18 and 90 years and (2) provided informed consent.

The exclusion criteria included the following: (1) hyperthyroidism or hypothyroidism; (2) secondary dyslipidemia or abnormal blood pressure/glucose levels; (3) type 1 diabetes mellitus; (4) use of glucocorticoids, contraceptives, weight-loss medications, or other drugs affecting body weight; (5) pregnancy or lactation; (6) acute infection; (7) severe heart, liver, or kidney dysfunction (defined by a serum creatinine clearance rate <30 mL/min or alanine aminotransferase ≥2.5 times the upper limit of normal values along with total bilirubin ≥1.5 times the upper limit of normal values in chronic heart failure patients classified as New York Heart Association functional class III or higher); and (8) malignancy.

During participant selection for this study, we further excluded specific categories on the basis of the following criteria:

(1)  Incomplete data regarding neutrophil counts, lymphocyte counts, platelet counts, or vitamin D levels in 212 participants;
(2)  Incomplete lifestyle information—including smoking status, alcohol consumption habits, and duration of sunlight exposure during the summer months—resulting in the exclusion of an additional 222 participants;
(3)  Extreme results for laboratory parameters such as complete blood count, glucose, lipid profiles, and uric acid levels, leading to the exclusion of 10 participants;
(4)  Extreme leukocyte counts (>50 × 10^9^/L or <1.0 × 10^9^/L), thereby eliminating potential confounding effects related to acute infection on inflammatory status.

Ultimately, 726 participants were included after screening.

### Laboratory tests

Blood samples were collected from all participants after fasting for a minimum period of 8 h on the subsequent morning.

- Serum 25-hydroxyvitamin D [25(OH)D]concentration was measured using an electrochemiluminescence immunoassay (ECLIA) with reagents provided by Roche Diagnostics Products (Shanghai) Co., Ltd. The assay demonstrated high precision, with intra-assay CV ranging from 1.3 to 3.0% and inter-assay CV ranging from 1.5 to 4.6%.
- The enumeration of platelet neutrophil lymphocytes was performed via an XN1000 hematology analyser employing whole blood cell counting methodology.
- Samples were analysed via a Cobas 8000 biochemistry analyser, which measures various parameters, including fasting glucose, total cholesterol triglycerides, high-density lipoprotein cholesterol, low-density lipoprotein cholesterol and uric acid concentrations, while glycated hemoglobin A1c levels were assessed with Sebia CAP instrumentation.

### Calculation of the systemic immune inflammatory index (SII)

The systemic immuno-inflammatory index (SII), initially conceived as a prognostic marker for patients following curative resection of hepatocellular carcinoma (30), represents an innovative scoring system predicated on the enumeration of lymphocytes, neutrophils, and platelets. This index delineates a heightened SII value as indicative of fluctuations in the levels of these three blood cell components, which are integral to both inflammatory and immune responses. The SII amalgamates these parameters through a calculated formula, thereby offering a more holistic representation of the patient’s inflammatory and immunological profile.

The SII is calculated according to the following formula (30):


SII=platelet count×neutrophil count/lymphocyte count

It is expressed as 1,000 cells per microliter.

### Vitamin D group classification

Following guidelines established by the American Endocrine Society in their publication dated 2011 (31), the participants were divided into three groups on the basis of their serum 25(OH)D levels.

Vitamin D deficiency group: (SVD < 20 ng/mL).
Vitamin D suboptimal group: (20 ng/mL ≤ SVD < 30 ng/mL).
Vitamin D optimal group: (SVD ≥ 30 ng/mL)

### Anthropometric measurements and questionnaire assessment

#### Body measurement

Body mass index (BMI) was calculated by dividing weight in kilograms (kg) by height squared in meters (m^2^), with measurements conducted by trained nursing professionals following standardized protocols.

#### Questionnaire survey

The sample data for this study were derived entirely from clinical case report forms (CCRFs). The participants completed these forms independently.

##### Structure of the questionnaire survey

The questionnaire was organized into several sections, including the following:

- Basic Information: Name, gender, age, etc.
- Health status: past medical history and family medical history.
- Lifestyle factors: Sleep patterns (normal sleep/sleep disorders/drowsiness; duration of nighttime sleep), alcohol consumption status (including whether the individual currently drinks and if they have quit, as well as weekly alcohol intake), smoking status (encompassing whether the individual smokes and if they have quit, along with average daily cigarette consumption), alcohol consumption habits (current drinking status and whether participants have quit drinking; weekly volume consumed), smoking behaviors (current smoking status and whether participants have quit smoking; average daily intake), exercise frequency and duration, sunlight exposure practices (active engagement and daily duration of exposure), and air conditioning usage during the summer months.
- Dietary habits: Preferences for warm versus cold foods; degree of preference for spicy or fatty foods; frequency of breakfast consumption; frequency of eating before bedtime; meat consumption frequency; intake frequency of sugary beverages and tea.

##### Methodology for conducting the questionnaire survey

Basic participant information was collected through face–to–face interviews to ensure accuracy.

##### Quality control measures for questionnaires

A comprehensive questionnaire was developed on the basis of the research objectives and principles guiding the survey process. A presurvey phase was implemented to identify potential issues within the questionnaire, allowing timely modifications and improvements.

All personnel involved in data collection possessed foundational medical knowledge and received training prior to conducting surveys to standardize methodologies aimed at minimizing bias throughout the investigation. For options that may pose comprehension challenges to respondents, the interviewers provided appropriate clarifications.

### Statistical analysis

The data were analysed via SPSS version 26.0. Continuous variables that conformed to a normal distribution are presented as the mean ± standard deviation (SD), with group comparisons conducted via one-way analysis of variance (ANOVA). For continuous variables not meeting the criteria for normality, the results are expressed as medians (interquartile ranges [IQRs]), and group differences were assessed with the Kruskal–Wallis H test. Categorical variables are reported as frequencies and percentages, with intergroup differences evaluated through chi-square tests or Fisher’s exact tests where appropriate. An ordered multinomial logistic regression analysis was employed to identify factors influencing vitamin D deficiency. The association between 25-hydroxyvitamin D [25(OH)D] and the systemic immune-inflammation index (SII) was examined via Spearman’s rank correlation coefficient. All the statistical tests were two-tailed, and a *p* value of <0.05 was considered to indicate statistical significance.

## Results

### Results of the study on participants’ basic characteristics

This study ultimately included a total of 726 participants, categorized into three groups according to their serum vitamin D levels: the optimal group (*n* = 329), the suboptimal group (*n* = 289), and the deficient group (*n* = 108). The sex distribution did not differ significantly among these groups (*p* > 0.05). However, age analysis indicated that the deficient group [55.00 (42.25, 66.00)] was significantly younger than both the optimal group [62.00 (54.00, 68.00)] and the suboptimal group [61.00 (50.50, 68.00)] (*p* < 0.05), as detailed in Table 1. Furthermore, there were no significant differences in past medical history—including diabetes, hypertension, and hyperlipidemia—across all groups (*p* > 0.05), as presented in Table 2.

**Table 1:** Demographic characteristics of populations stratified by serum vitamin D levels.

Demographic characteristics	Optimal group (*n* = 329)	Suboptimal group (*n* = 289)	Deficiency group (*n* = 108)	χ^2^/*Z*	*p* value
Sexual				0.038	0.981
Male	142 (43.16)	127 (43.94)	47 (43.52)		
Female	187 (56.84)	162 (56.06)	61 (56.48)		
Age	62.00 (54.00,68.00)	61.00 (50.50,68.00)	55.00 (42.25,66.00)ab	12.39	0.002**

The demographic characteristics of the participants categorized by their serum vitamin D levels were analysed. A total of 726 individuals (*n* = 768) were included in the study and were stratified into three groups on the basis of their serum vitamin D concentrations: the optimal group (*n* = 329), the suboptimal group (*n* = 289), and the deficient group (*n* = 108). Notably, the age of participants in the deficient group was significantly lower than that of both the optimal and suboptimal groups.

a: indicates significance compared with the optimal group (*p* < 0.05); b: indicates significance compared with the suboptimal group (*p* < 0.05). *p < 0.05, **p < 0.01; *p* < 0.05 was considered statistically significant.

**Table 2:** Presents the variations in medical history among populations stratified by serum vitamin D level.

History	Optimal group (*n* = 329)	Suboptimal group (*n* = 289)	Deficiency group (*n* = 108)	*χ* ^2^	*p* value
Diabetes	292 (88.75)	240 (83.04)	95 (87.96)	4.533	0.104
Hypertension	177 (53.80)	156 (53.98)	54 (50.00)	0.559	0.756
Dyslipidemia	171 (51.98)	166 (57.44)	63 (58.33)	2.394	0.302
Fatty liver	129 (39.21)	125 (43.25)	54 (50.00)	4.011	0.135
Gout/hyperuricemia	110 (33.43)	88 (30.45)	32 (29.63)	0.88	0.644
Osteoporotic condition	27 (8.21)	18 (6.23)	6 (5.56)	1.341	0.511
Osteoarthritic disease	14 (4.26)	11 (3.81)	3 (2.78)	0.354	0.869
Sleep apnea	8 (2.43)	6 (2.08)	6 (5.56)	3.474	0.180
Abdominal obesity	249 (75.68)	223 (77.16)	91 (84.26)	3.475	0.176

No significant differences were observed in past medical history across the groups (*p* > 0.05).

*p < 0.05, **p < 0.01; *p* < 0.05 was considered statistically significant.

### Biochemical indicator comparison

The vitamin D-deficient group exhibited adverse presented negative trends in several metabolic indicators. Compared with the other two groups, this group presented significantly elevated levels of hemoglobin A1c [8.20 (6.72, 10.60)%] and fasting blood glucose [7.56 (6.01, 9.68) mmol/L] (*p* < 0.05). With respect to lipid profiles, the deficient group had markedly higher levels of triglycerides [1.67 (1.21, 2.63) mmol/L] and low-density lipoprotein cholesterol [3.29 (2.47, 3.94) mmol/L] than did the optimal group (*p* < 0.05), whereas high-density lipoprotein cholesterol levels were significantly lower in the deficient group [1.04 (0.87, 1.27) mmol/L] than in the optimal group (*p* < 0.05). Furthermore, this group presented significantly increased levels of uric acid [383.00 (301.50, 469.75) μmol/L] and body mass index (BMI) [26.37 (23.15, 28.72) kg/m^2^] relative to those of the optimal group (*p* < 0.05). The details are presented in Table 3.

**Table 3:** Variations in biochemical markers among populations stratified by serum vitamin D levels.

Tests and measures	Optimal group (*n* = 329)	Suboptimal group (*n* = 289)	Deficiency group (*n* = 108)	*Z/F*	*p* value
Glycated hemoglobin	7.00 (6.20,8.20)	7.20 (6.10,8.85)	8.20 (6.72,10.60)ab	17.636	0.000**
Fasting plasma glucose	6.82 (5.87,8.30)	6.80 (5.66,8.72)	7.56 (6.01,9.68)ab	7.32	0.026*
Uric acid	353.00 (296.00,429.50)	383.00 (319.50,434.00)	383.00 (301.50,469.75)a	8.507	0.014*
Total cholesterol	4.65 (3.92,5.46)	4.72 (4.10,5.55)	5.00 (4.01,5.66)	5.702	0.058
Triglycerides	1.38 (1.01,2.08)	1.56 (1.04,2.23)	1.67 (1.21,2.63)a	11.621	0.003**
Low-density lipoprotein C (LDL-C)	2.87 (2.23,3.60)	3.14 (2.50,3.77)a	3.29 (2.47,3.94)a	13.439	0.001**
High-density lipoprotein C (HDL-C)	1.14 (0.98,1.37)	1.08 (0.92,1.32)a	1.04 (0.87,1.27)a	13.113	0.001**
Leukocyte count	6.81 (5.62,7.93)	6.72 (5.92,7.80)	7.03 (5.95,8.42)	4.398	0.111
Platelet count	234.00 (205.50,276.50)	242.00 (200.00,279.50)	246.00 (214.00,293.75)	2.781	0.249
Neutrophil count	4.07 (3.17,4.78)	3.95 (3.29,4.87)	4.27 (3.37,5.54)	5.605	0.061
Lymphocyte count	1.99 (1.63,2.44)	1.95 (1.60,2.50)	1.96 (1.58,2.34)	0.758	0.685
Monocyte count	0.48 (0.38,0.59)	0.46 (0.37,0.55)	0.44 (0.35,0.59)	4.365	0.113
Hemoglobin	136.71 ± 14.91	136.58 ± 16.13	135.31 ± 19.31	0.322	0.725
BMI	24.80 (22.70,26.98)	25.04 (22.89,27.47)	26.37 (23.15,28.72)a	8.891	0.012*

Notably, compared with those in both the optimal and suboptimal groups, the glycated hemoglobin and fasting blood glucose levels in the deficient group were significantly elevated (*p* < 0.05). Additionally, triglyceride and low-density lipoprotein cholesterol levels in the deficient group were significantly higher than those in the optimal group (*p* < 0.05), whereas high-density lipoprotein cholesterol levels were significantly lower (*p* < 0.05). Furthermore, uric acid and BMI levels in the deficient group were markedly higher than those in the optimal group (*p* < 0.05).

a: indicates significance compared with the optimal group (*p* < 0.05); b: indicates significance compared with the suboptimal group (*p* < 0.05). *p < 0.05, **p < 0.01; *p* < 0.05 was considered statistically significant.

### Comprehensive analysis of lifestyle and dietary habits

An analysis of the differences in lifestyle and dietary habits among the three groups was performed, with adjustments for confounding factors prior to conducting multiple comparisons. The findings indicated that the vitamin D-deficient group presented unhealthy trends across several lifestyle and dietary practices. Compared with the other two groups, this group was more likely to engage in late-night eating, consume sugary beverages more frequently, prefer cold foods, and spend more hours using air conditioning during the summer [10.00 (6.00, 15.00) hours/day] (*p* < 0.05) while exhibiting reduced sunlight exposure [0.00 (0.00, 18.75) hours/day] (*p* < 0.05). Furthermore, relative to the optimal group, the deficient group reported a higher work stress score [6.00 (4.00, 8.00)] (*p* < 0.05), as detailed in Table 4.

**Table 4:** Variations in lifestyle and dietary practices among populations stratified by serum vitamin D levels.

Lifestyle factors	Optimal group (*n* = 329)	Suboptimal group (*n* = 289)	Deficiency group (*n* = 108)	*X* ^2^ */Z*	*p* value
Dietary preferences	0.00 (0.00,1.00)	0.00 (0.00,1.00)	0.00 (0.00,1.00)	4.173	0.124
Consuming breakfast	7.00 (7.00,7.00)	7.00 (7.00,7.00)	7.00 (7.00,7.00)a	7.497	0.024*
Consuming meals before sleep	0.00 (0.00,1.00)	0.00 (0.00,0.00)	0.00 (0.00,3.00)ab	14.884	0.001**
Frequency of meat intake	7.00 (7.00,7.00)	7.00 (7.00,7.00)	7.00 (7.00,7.00)	0.172	0.918
Frequency of consuming sugary drinks	0.00 (0.00,0.00)	0.00 (0.00,0.00)	0.00 (0.00,1.75)ab	29.014	0.000**
Frequency of consuming tea	3.00 (0.00,7.00)	3.00 (0.00,7.00)	2.50 (0.00,7.00)	0.922	0.631
Frequency of consuming cold and raw foods	0.00 (0.00,0.00)	0.00 (0.00,1.00)	0.00 (0.00,2.00)a	12.14	0.002**
Work stress score	0.00 (0.00,1.00)	0.00 (0.00,3.00)	0.00 (0.00,4.00)a	18.204	0.000**
Average daily hours of sun exposure	0.00 (0.00,30.00)	0.00 (0.00,30.00)	0.00 (0.00,18.75)ab	12.315	0.002**
Daily hours of air conditioning in summer	8.00 (6.00,10.00)	8.00 (5.00,12.00)	10.00 (6.00,15.00)ab	9.600	0.008**
Smoking				11.964	0.054
No	248 (75.38)	234 (80.97)	88 (81.48)		
Yes	40 (12.16)	38 (13.15)	16 (14.81)		
Secondhand smoke exposure	7 (2.13)	3 (1.04)	1 (0.93)		
Quit smoking	34 (10.33)	14 (4.84)	3 (2.78)		
Drinking alcohol				3.933	0.412
No	275 (83.59)	254 (87.89)	94 (87.04)		
Yes	40 (12.16)	25 (8.65)	8 (7.41)		
Quit drinking alcohol	14 (4.26)	10 (3.46)	6 (5.56)		
Sleeping condition				4.567	0.271
Normal	196 (59.57)	187 (64.71)	63 (58.33)		
Sleep disorders	132 (40.12)	102 (35.29)	44 (40.74)		
Lethargy	1 (0.30)	0 (0.00)	1 (0.93)		
Appetite				11.273	0.070
Normal	265 (80.55)	236 (81.66)	84 (77.78)		
Hunger without desire for food	6 (1.82)	6 (2.08)	0 (0.00)		
Overeating often leads to frequent hunger	40 (12.16)	21 (7.27)	17 (15.74)		
Appetite loss	18 (5.47)	26 (9.00)	7 (6.48)		
Prefer warm	27 (8.21)	35 (12.11)	13 (12.04)	2.93	0.231
Prefer cold	6 (1.82)	10 (3.46)	9 (8.33)a	9.012	0.009**
Active movement	235 (71.43)	196 (67.82)	66 (61.11)	4.099	0.129
The maximum daily duration of sedentary behavior				29.264	0.001**
< 1 h	96 (29.18)	80 (27.68)	16 (14.81)ab		
1–2 h	120 (36.47)	88 (30.45)	41 (37.96)		
2–4 h	65 (19.76)	45 (15.57)	22 (20.37)		
4–6 h	23 (6.99)	38 (13.15)a	13 (12.04)		
6–8 h	19 (5.78)	22 (7.61)	6 (5.56)		
> 8 h	6 (1.82)	16 (5.54)a	10 (9.26)a		

After adjusting for confounding factors, the results indicated that the deficient group presented a greater frequency of prebedtime eating, consumption of sugary beverages, preference for cold foods, increased duration of air conditioning use during the summer (*p* < 0.05), and reduced sunlight exposure time (*p* < 0.05). Additionally, the work stress scores of the deficient group were significantly greater than those of the optimal group (*p* < 0.05).

a: indicates significance compared with the optimal group (*p* < 0.05); b: indicates significance compared with the suboptimal group (*p* < 0.05). *p < 0.05, **p < 0.01; *p* < 0.05 was considered statistically significant.

### Risk factors for vitamin D deficiency

The significant variables from the univariate analysis were identified to serve as independent variables, and an ordered multivariate logistic regression was performed with vitamin D deficiency groups designated as the dependent variable. There was no evidence of severe multicollinearity among the independent variables (all VIF values<5), and the model demonstrated an acceptable goodness-of-fit (X^2^ = 1458.557, *p* = 0.313 > 0.05). The validity of the model was confirmed through a parallel line test (*p* = 0.129 > 0.05), which satisfies the assumption of a “proportional advantage.” The results of the multivariate logistic regression analysis indicated that the frequency of sugary beverage consumption (OR = 1.225, 95% CI: 1.074–1.398), work stress score (OR = 1.136, 95% CI: 1.025–1.259), hemoglobin A1c level (OR = 1.126, 95% CI: 1.038–1.221), low-density lipoprotein cholesterol level (OR = 1.243, 95% CI: 1.074–1.439), and body mass index (BMI) (OR = 1.044, 95% CI: 1.002–1.088) were identified as independent risk factors for vitamin D deficiency. Conversely, sunlight exposure duration (OR = 0.996, 95% CI: 0.993–0.999) was the sole protective factor. The details are presented in Table 5.

**Table 5:** Logistic regression analysis of serum vitamin D deficiency risk factors.

Variable	B	SE	Wald	*p* value	OR	95%CI		VIF
						Lower limit	Upper limit	
Age	0.006	0.008	0.588	0.443	1.006	0.991	1.022	1.951
Consuming breakfast	0.012	0.070	0.027	0.869	1.012	0.882	1.161	1.160
Consume meals before sleep	0.007	0.036	0.037	0.848	1.007	0.938	1.081	1.056
Frequency of consuming sugary drinks	0.203	0.067	9.089	0.003**	1.225	1.074	1.398	1.407
Frequency of consuming cold and raw foods	0.054	0.059	0.833	0.361	1.055	0.940	1.185	1.413
Work stress score	0.128	0.053	5.861	0.015*	1.136	1.025	1.260	1.830
Average daily hours of sun exposure	−0.004	0.002	6.048	0.014*	0.996	0.993	0.999	1.035
Daily hours of air conditioning in summer	−0.005	0.014	0.137	0.711	0.995	0.969	1.022	1.284
Glycated hemoglobin	0.119	0.040	8.733	0.003**	1.126	1.041	1.218	1.431
Fasting plasma glucose	−0.016	0.030	0.273	0.601	0.984	0.927	1.045	1.390
Uric acid	0.001	0.001	0.967	0.326	1.001	0.999	1.002	1.211
Triglycerides	0.061	0.062	0.988	0.320	1.063	0.942	1.200	1.194
Low-density lipoprotein C (LDL-C)	0.218	0.074	8.543	0.003**	1.243	1.074	1.439	1.079
High-density Lipoprotein C (HDL-C)	0.180	0.144	1.563	0.211	1.197	0.903	1.588	1.083
BMI	0.043	0.021	4.241	0.039*	1.044	1.002	1.088	1.232
The maximum daily duration of sedentary behavior	0.100	0.055	3.310	0.069	1.105	0.992	1.231	1.118
Prefer cold and warm food								
Yes					1.000			
No	−0.421	0.421	1.000	0.317	0.657	0.288	1.498	1.113

Significant variables identified from the univariate analysis were selected as independent variables, with vitamin D deficiency categorized as the dependent variable for ordered multivariate logistic regression. The covariates included the frequency of sugary beverage consumption, work stress score, hemoglobin A1c, low-density lipoprotein cholesterol, and BMI. The model successfully passed the parallel line test and met the “proportional odds” assumption. The results indicated that the frequency of sugary drink intake, work stress score, hemoglobin A1c level, low-density lipoprotein cholesterol level, and BMI are independent risk factors for vitamin D deficiency; conversely, sunlight exposure duration emerged as a significant protective factor.

*p < 0.05, **p < 0.01; *p* < 0.05 was considered statistically significant.

### Differences in the SII

**Figure 1:**
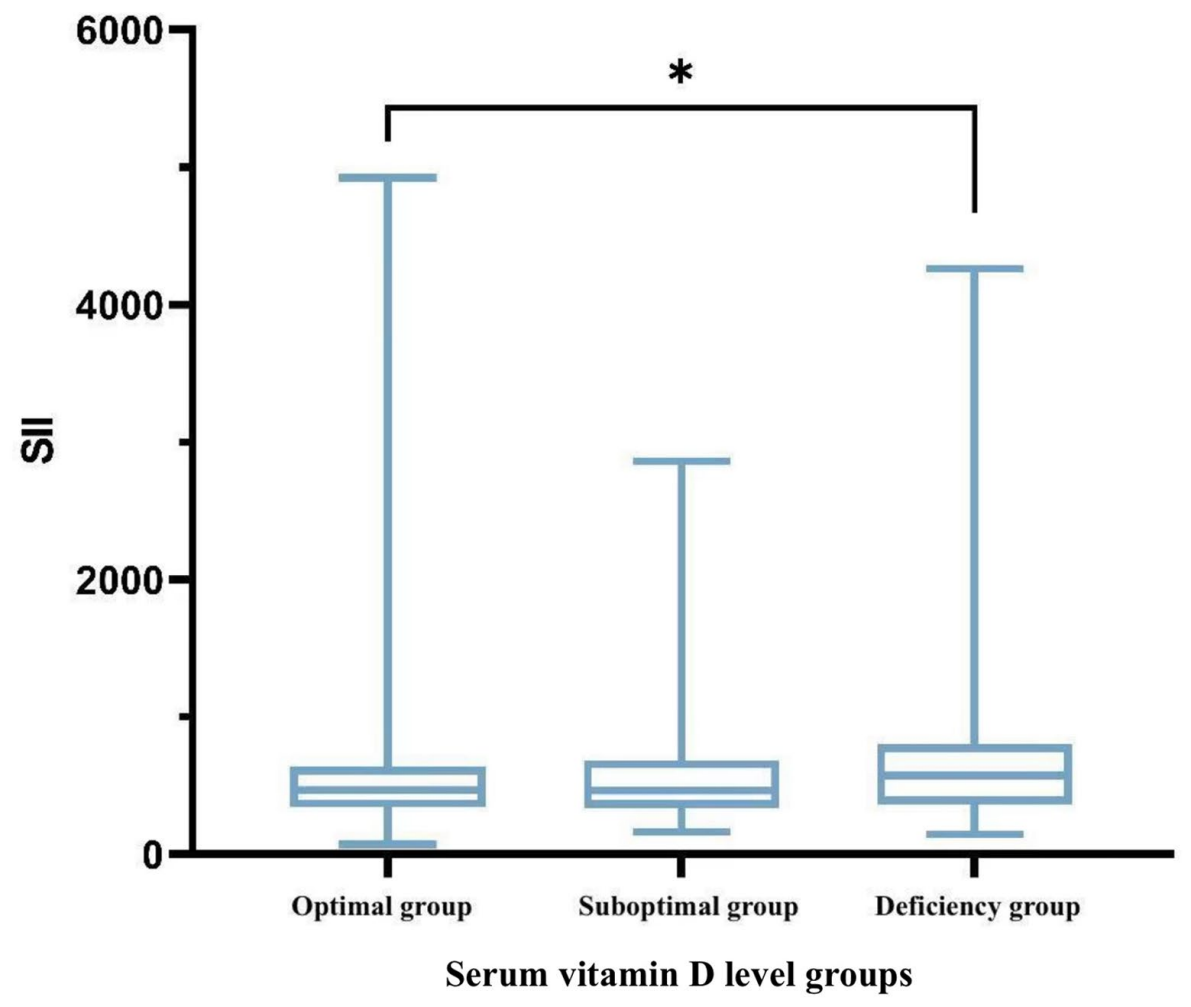
Boxplot illustrating the variability of SII across different groups. *p < 0.05, **p < 0.01; *p* < 0.05 was considered statistically significant.

Significant differences in the SII were observed among the three groups (*p* < 0.05). The SII in the vitamin D-deficient group [575.47 (363.23, 802.89)] was markedly higher than that in the optimal group [468.94 (340.79, 639.20)] (*p* < 0.05), suggesting that lower vitamin D levels may be linked to an elevated systemic inflammatory state, as illustrated in Table 6 and Figure 1.

**Table 6:** Variations in the SII among populations stratified by serum vitamin D levels.

	Optimal group (*n* = 329)	Suboptimal group (*n* = 289)	Deficiency group (*n* = 108)	*Z*	*p* value
SII	468.94 (340.79,639.20)	466.28 (339.60,685.86)	575.47 (363.23,802.89)a	7.459	0.024*

Significant differences in the SII were observed among the three groups (*p* < 0.05). The SII in the vitamin D-deficient group was markedly higher than that in the optimal group (*p* < 0.05). a: indicates significance compared with the optimal group (*p* < 0.05). *p < 0.05, **p < 0.01; *p* < 0.05 was considered statistically significant.

### Age-stratified analysis of the association between vitamin D levels and the SII

**Figure 2:**
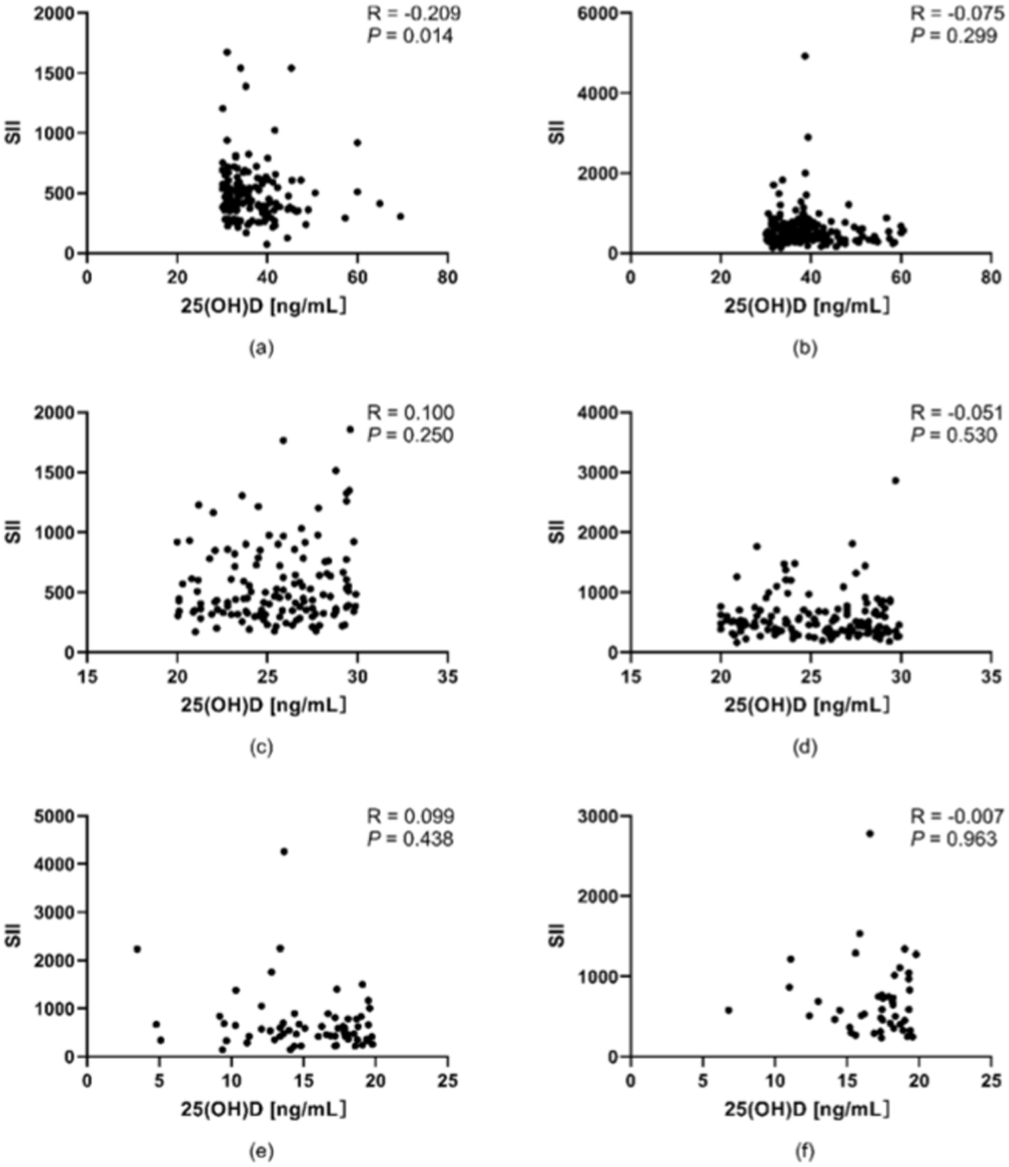
Associations between vitamin D levels and the SII across various age groups. **(a)** Young and middle-aged people in the optimal group. **(b)** Old people in the optimal group. **(c)** Young and middle-aged people in the suboptimal group. **(d)** Old people in the suboptimal group. **(e)** Young and middle-aged people in the deficient group. **(f)** Elderly people in the deficient group. A significant negative correlation was identified between vitamin D levels and the SII in the optimal cohort of young and middle-aged individuals (r = 0.209, *p* < 0.05). *p < 0.05, **p < 0.01; *p* < 0.05 was considered statistically significant.

Following the stratification of participants into a young-to-middle-aged group (<60 years) and an elderly group (≥60 years), the correlation between 25(OH)D levels and the SII was assessed with Spearman’s rank correlation coefficient on the basis of age categories. A significant negative correlation was identified exclusively in the optimal subgroup of young-to-middle-aged individuals (r = −0.209, *p* < 0.05). This finding indicates that the association between vitamin D levels and inflammatory status may vary with age, as illustrated in Figure 2.

### Stratified analysis of the association between vitamin D levels and the SII by BMI

**Figure 3:**
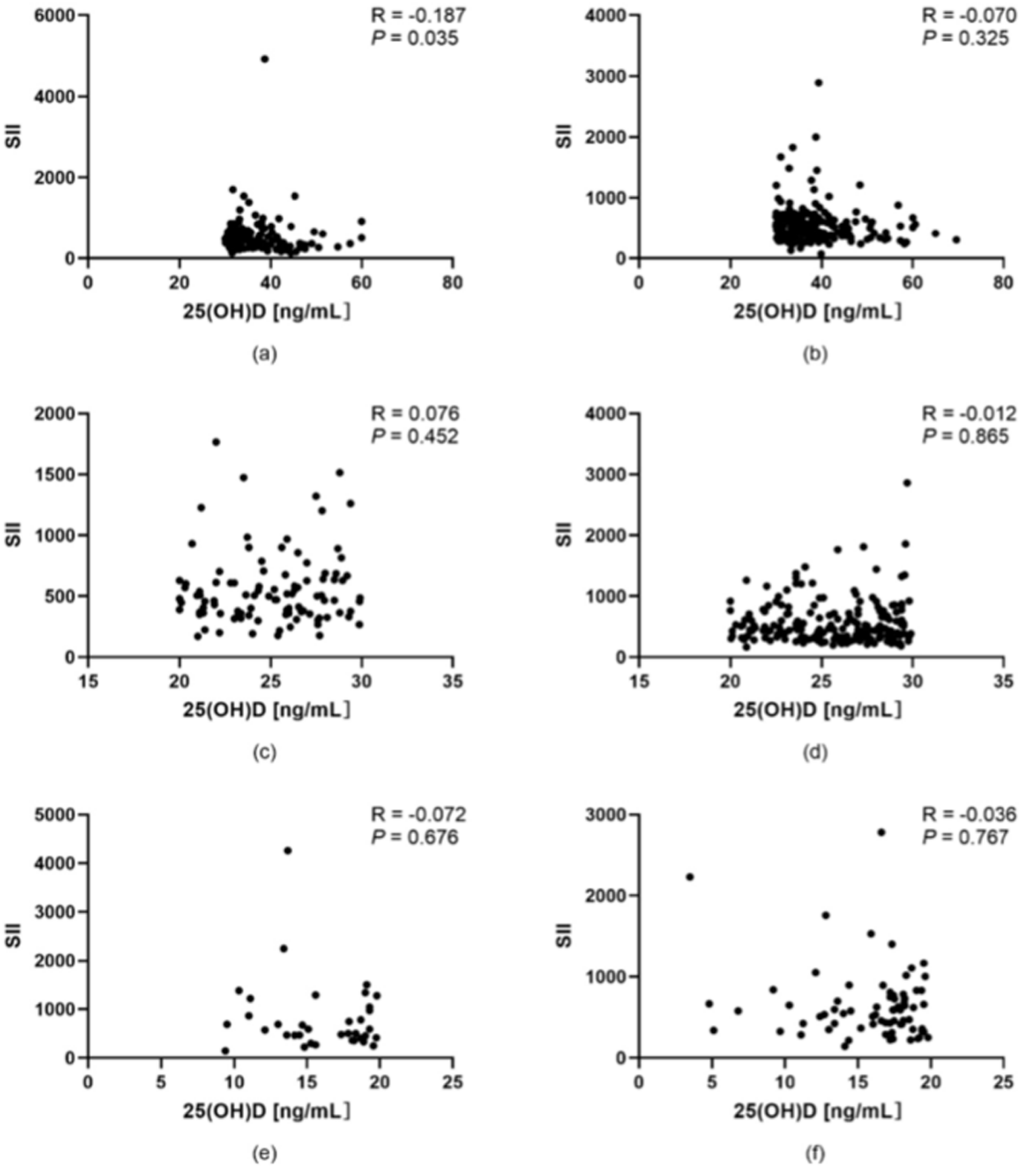
Correlation between vitamin D levels and the SII in populations stratified by BMI. **(a)** Participants with BMI < 24.0 in the optimal vitamin D group. **(b)** Participants with BMI ≥ 24.0 in the optimal vitamin D group. **(c)** Participants with BMI < 24.0 in the suboptimal vitamin D group. **(d)** Participants with BMI ≥ 24.0 in the suboptimal vitamin D group. **(e)** Participants with BMI < 24.0 in the vitamin D deficiency group. **(f)** Participants with BMI ≥ 24.0 in the vitamin D deficiency group. *p < 0.05, **p < 0.01; *p* < 0.05 was considered statistically significant.

According to the World Health Organization (WHO), an adult with a BMI ≥ 25 is classified as overweight. However, based on the latest “Obesity Diagnosis and Treatment Guidelines (2024 Edition)” and “Weight Management Guidelines (2024 Edition)” in China, the BMI classification for Chinese adults is as follows: underweight (BMI < 18.5), normal weight (BMI 18.5–23.9), overweight (BMI 24.0–27.9), and obese (BMI ≥ 28.0). Therefore, in this study, participants were stratified into two groups (BMI < 24 and BMI ≥ 24) to analyze the correlation between vitamin D levels and SII. The Spearman rank correlation coefficient was used to assess the association between 25(OH)D levels and SII. As shown in Figure 3, in the optimal vitamin D group, there was a significant negative correlation between 25(OH)D levels and SII among subjects with BMI < 24.0 (*p* < 0.05; correlation coefficient = −0.187, indicating a weak correlation). In contrast, no significant correlation was observed between 25(OH)D levels and SII among subjects with BMI ≥ 24.0 in the same group (*p* > 0.05). Similarly, in the suboptimal vitamin D group and the vitamin D deficiency group, no significant correlations were found between 25(OH)D levels and SII across either BMI stratum (*p* > 0.05 for all comparisons). For detailed results, as illustrated in Figure 3.

### Independent and interactive effects of vitamin D levels and lifestyle factors on the SII

This study systematically evaluated the independent and interactive effects of serum 25(OH)D concentration and lifestyle factors on the SII using a stratified regression model, while adjusting for potential confounding factors (gender, age). No severe multicollinearity was observed among the independent variables (all VIF values<5). Model 1 demonstrates that the frequency of sugary beverage intake is positively associated with SII (B = 32.189, *p* < 0.05), whereas 25(OH)D exhibits a significant negative association with SII (B = −4.248, *p* < 0.05). Neither daily sun exposure time nor work stress score showed significant associations with SII (*p* > 0.05). After incorporating interaction terms, Model 2 reveals that the interaction between 25(OH)D and sugary beverage intake significantly negatively affects SII (B = −3.143, *p* < 0.05). However, the interactions involving daily sun exposure time and 25(OH)D, as well as work stress score and 25(OH)D, were not statistically significant (*p* > 0.05). Model 3, refined from Model 2 by excluding non-significant interaction terms, indicates that the frequency of sugary beverage intake does not significantly affect SII (B = 12.944, *p* > 0.05), while 25(OH)D continues to exhibit a significant negative association with SII (B = −4.280, *p* < 0.05). Additionally, the interaction term between 25(OH)D and sugary beverage intake significantly negatively influences SII (B = −2.960, *p* < 0.05). These findings suggest that higher sugary beverage intake frequency may amplify the protective effect of vitamin D on SII. For detailed results, as illustrated in Table 7.

**Table 7:** Independent and interactive effects of serum 25(OH)D concentration and lifestyle factors on the SII.

Variable	Model 1			Model 2			Model 3		
	B	*t*	*p*	B	*t*	*p*	B	*t*	*p*
Frequency of consuming sugary drinks	31.971	2.573	0.010*	12.596	0.865	0.387	12.944	0.891	0.373
Average daily hours of sun exposure	−0.127	−0.431	0.666	−0.079	−0.260	0.795			
Work stress score	−3.545	−0.324	0.746	−2.299	−0.209	0.835			
25(OH)D	−4.198	−2.649	0.008**	−4.177	−2.642	0.008**	−4.280	−2.725	0.007**
Frequency of consuming sugary drinks × 25(OH)D				−3.021	−2.552	0.011*	−2.960	−2.515	0.012*
Average daily hours of sun exposure × 25(OH)D				−0.013	−0.547	0.584			
Work stress score × 25(OH)D				0.494	0.544	0.587			

This study systematically evaluated the independent and interactive effects of serum 25(OH)D concentration and lifestyle factors on the SII using a stratified regression model, while adjusting for potential confounding factors (gender, age). Model 1 demonstrates that the frequency of sugary beverage intake is positively associated with SII (B = 32.189, *p* < 0.05), whereas 25(OH)D exhibits a significant negative association with SII (B = −4.248, *p* < 0.05). Neither daily sun exposure time nor work stress score showed significant associations with SII (*p* > 0.05). After incorporating interaction terms, Model 2 reveals that the interaction between 25(OH)D and sugary beverage intake significantly negatively affects SII (B = −3.143, *p* < 0.05). However, the interactions involving daily sun exposure time and 25(OH)D, as well as work stress score and 25(OH)D, were not statistically significant (*p* > 0.05). Model 3, refined from Model 2 by excluding non-significant interaction terms, indicates that the frequency of sugary beverage intake does not significantly affect SII (B = 12.944, *p* > 0.05), while 25(OH)D continues to exhibit a significant negative association with SII (B = −4.280, *p* < 0.05). Additionally, the interaction term between 25(OH)D and sugary beverage intake significantly negatively influences SII (B = −2.960, *p* < 0.05). These findings suggest that higher sugary beverage intake frequency may amplify the protective effect of vitamin D on SII.

*p < 0.05, **p < 0.01; *p* < 0.05 was considered statistically significant.

## Discussion

This study investigated the associations between serum 25-hydroxyvitamin D (25(OH)D) levels and the systemic immune-inflammatory index (SII), metabolic parameters, and dietary lifestyle habits in a cohort of 726 adults with varying vitamin D statuses. The results elucidate the intricate associations among vitamin D, chronic inflammation, metabolic health, and lifestyle choices, thereby offering novel insights into the significance of vitamin D within public health contexts.

### The association between vitamin D and the systemic inflammatory immune response

The findings of this study support our hypothesis, demonstrating a significant difference in the systemic immune-inflammatory index (SII) among individuals with varying vitamin D levels. Specifically, those classified as vitamin D deficient (SVD < 20 ng/mL) presented a markedly higher SII than individuals with optimal vitamin D levels (SVD ≥ 30 ng/mL), indicating that lower vitamin D levels are associated with increased systemic inflammation. These results align with the literature. The findings of a study focused on adolescents revealed that serum TNFR-2 levels were significantly elevated in individuals with severe vitamin D deficiency (SVD < 10 ng/mL) and exhibited a significant negative correlation with vitamin D levels across the overall sample (32). Similarly, another investigation in American adults revealed that CRP levels were elevated in the SVD < 10 ng/mL cohort relative to those in the SVD ≥ 30 ng/mL cohort (33). These studies collectively suggest that individuals with severe vitamin D deficiency (SVD < 10 ng/mL) exhibit elevated levels of inflammation within the general population. Our study, building upon this foundation, also demonstrated that individuals with vitamin D levels ranging from 10 ng/mL to 20 ng/mL exhibit similarly elevated inflammatory levels. This finding suggests that, in addition to those with severe vitamin D deficiency, there may be a risk of chronic inflammation when vitamin D levels fall below 20 ng/mL. Such insights could have significant implications for disease prevention in clinical practice. Currently, the only available study examining the association between vitamin D and the SII reveals a significant correlation between the SII and serum 25(OH)D level in patients suffering from ischaemic heart disease, particularly those with acute coronary syndrome (24). Our findings corroborate these results within a broader population context. Furthermore, animal studies have demonstrated that supplementation with vitamin D can reduce the mRNA expression of CD11c, CD68, and iNOS, which are specific to inflammatory M1 macrophages, while lowering serum NO concentrations and significantly reducing the expression of proinflammatory genes, such as IL-6, MCP-1, and TNF-*α* (34). These findings provide additional evidence regarding the role of vitamin D in modulating inflammatory processes.

Furthermore, this study revealed that the SII did not significantly change when SVD levels were within the range of 20–30 ng/mL. We hypothesize the existence of a threshold effect, with the threshold point identified in this study at 20 ng/mL. Below this vitamin D level, there is a significant increase in systemic inflammation. However, when vitamin D levels exceed 20 ng/mL, within a specific concentration range, further increases in vitamin D may not demonstrate a significant association with inflammatory markers. Nonetheless, the current literature lacks definitive studies delineating this specific interval; thus, additional research is warranted to validate these findings.

In addition, we performed a stratified analysis based on BMI. The results indicated that in individuals with serum vitamin D (SVD) levels≥30 ng/mL and BMI < 24, 25(OH)D exhibited a weak negative correlation with the SII (*p* < 0.05). This suggests that under conditions of normal body weight and sufficient vitamin D status, vitamin D may exert a certain regulatory effect on systemic inflammation. However, in all subgroups with BMI ≥ 24.0 or SVD < 30 ng/mL, no significant correlation was observed between 25(OH)D and SII (*p* > 0.05). This finding may be attributed to metabolic disturbances and increased inflammatory factor release associated with obesity, which could obscure the effects of vitamin D. Additionally, it highlights the limited capacity of vitamin D to regulate immune function when its levels are insufficient. Vitamin D may influence systemic inflammation through various mechanisms, including modulating immune cell function, regulating inflammatory cytokine secretion, and protecting vascular endothelial integrity, particularly when body weight is within the normal range and vitamin D levels are adequate.

Although the Endocrine Society’s forthcoming guidelines were published in 2024, they no longer designate a target level of 25(OH)D at 30 ng/mL and do not classify vitamin D thresholds (2). Our findings indicate that maintaining elevated levels of vitamin D may positively influence the suppression of chronic inflammation among Chinese adults, particularly middle-aged and young adults. This insight is crucial for informing public health strategies and clinical guidelines, especially regarding approaches to mitigate the risk of diseases associated with chronic inflammation through vitamin D supplementation. Furthermore, it underscores the necessity of considering individual variability—including factors such as race, geographic location, and lifestyle—when implementing these guidelines, as these elements can significantly impact an individual’s requirement for vitamin D.

### Mechanism of the association of vitamin D with chronic inflammation

The association between vitamin D and chronic inflammation is multifaceted, with the current understanding suggesting that vitamin D regulates the immune system through various pathways to exert its anti-inflammatory effects. The expression of vitamin D receptor (VDR) genes is prevalent across a range of immune cells (35–38). 1,25-Dihydroxyvitamin D [1,25(OH)2D] interacts with the vitamin D receptor (VDR) to modulate the immune response of immune cells, thereby significantly influencing the onset and progression of chronic inflammation.

First, 1,25(OH)2D has regulatory effects on macrophages and dendritic cells by promoting the differentiation of monocytes into macrophages while simultaneously reducing their production of proinflammatory cytokines and chemokines. Experimental studies have demonstrated that 1,25(OH)2D can inhibit the expression of innate immune receptors such as TLR2, TLR4, and TLR9 and affect the synthesis of inflammatory cytokines such as IL-6 (39). Additionally, 1,25(OH)2D downregulates cell surface MHC antigen expression (40), which plays a crucial role in mediating immune responses and regulating certain pathological conditions. Furthermore, vitamin D inhibits the proliferation, differentiation, and maturation of dendritic cells; it also regulates lymphocyte immunoglobulin stability and production (including T cells and B cells); suppresses Th1 and Th17 proinflammatory responses; and promotes Tr1 (IL-10), Th2 (IL-4, IL-5 and IL-10) (41), and anti-inflammatory regulatory T-cell (T-reg) activation (42, 43), thereby reducing the levels of proinflammatory cytokines such as IL-1 and TNF (20, 21). The actions that balance proinflammatory and anti-inflammatory cytokines are primarily mediated through the inhibition of the NF-κB pathway by the vitamin D receptor (VDR) (22). Additionally, vitamin D regulates the expression of Toll-like receptors (TLR-2 and TLR-4) (39) and modulates neutrophil function. Research indicates that vitamin D can inhibit the production of proinflammatory mediators and reactive oxygen species (ROS) in neutrophils, thereby mitigating inflammation (44). In a separate study involving women, the level of 25(OH)D was positively correlated with leukocyte telomere length, which is a marker indicative of biological aging, oxidative stress elevation, and chronic inflammation (44, 45). These findings further underscore that sufficient vitamin D levels are associated with reduced chronic inflammation.

In addition to its effects on lymphocytes and leukocytes in relation to chronic inflammation, vitamin D also plays a role in platelet activation and aggregation—processes closely linked to inflammatory responses (46). Platelets not only contribute to hemostasis but also actively participate in immune responses (47, 48). A study by Ebin Johny et al. demonstrated that activated platelets secrete substantial amounts of inflammatory mediators. Moreover, platelet immune cells are critically involved in the chronic low-grade inflammation associated with type 2 diabetes and coronary artery disease (49). Another controlled clinical trial conducted by their team suggested that vitamin D supportive therapy may effectively reduce or prevent disease progression and cardiovascular risk in patients with type 2 diabetes mellitus (T2DM) through the inhibition of oxidative stress and platelet-mediated inflammation (50).

### The association between vitamin D and metabolic function

This study elucidates the significant associations between vitamin D levels and various metabolic factors. Using 20 ng/mL as the critical threshold for vitamin D deficiency, we observed that individuals with vitamin D levels below this threshold presented significantly elevated hemoglobin A1c and fasting blood glucose levels compared with those with levels equal to or above this critical value. In terms of lipid metabolism, a decrease in the vitamin D concentration was correlated with a decreasing trend in high-density lipoprotein (HDL) levels; additionally, triglyceride levels and BMI were higher in the deficient group than in those with vitamin D concentrations of 30 ng/mL or greater. Individuals with vitamin D level less than 30 ng/mL also presented increased low-density lipoprotein (LDL) levels, suggesting that maintaining higher vitamin D concentrations (particularly ≥30 ng/mL) may be more effective for regulating glucose metabolism, triglycerides, LDL cholesterol, and obesity. Furthermore, hemoglobin A1c, LDL cholesterol, and BMI emerged as independent risk factors associated with vitamin D status. These findings align with those of a study conducted in American adults, which indicated that individuals with SVD < 12 ng/mL presented significantly greater waist circumference, HbA1c levels, and triglycerides, as well as reduced HDL levels, as well as an elevated risk of metabolic syndrome and diabetes, than those with SVD ≥ 30 ng/mL (35). A recent study indicated that vitamin D enhances the probability of normalizing blood glucose regulation by 30%. Furthermore, among individuals with a 25(OH)D level of 50 ng/mL or higher, the risk of developing new-onset diabetes was reduced by 76% compared with that in individuals with 25(OH)D levels ranging from 20 to 29 ng/mL (25). The Endocrine Society also underscores the beneficial effects of vitamin D supplementation in prediabetic patients (2). Collectively, our results suggest that the risk of developing diabetes is lower when SVD is equal to or greater than 20 ng/mL; additionally, maintaining SVD at or above 30 ng/mL confers significant benefits in mitigating hyperlipidemia and obesity.

The role of vitamin D in inhibiting NF-κβ activity represents a crucial mechanism in the regulation of blood glucose levels. It directly stimulates insulin secretion from pancreatic beta cells (51) and mitigates systemic inflammation by engaging vitamin D receptors located on pancreatic beta cells, skeletal muscle, and liver tissues (52), thereby alleviating insulin resistance. Additionally, vitamin D downregulates parathyroid hormone levels to counteract its inhibitory effects on insulin secretion (53). Collectively, these actions underscore the significant role of vitamin D in blood glucose regulation and its close association with lipid profiles (54).

The association between vitamin D and lipid metabolism is intricately linked to the consequences of vitamin D deficiency, which results in calcium exudation from adipocytes, thereby stimulating hyperparathyroidism and promoting lipogenesis (55). Additionally, vitamin D deficiency elevates fatty acid synthase levels (56), reduces leptin concentrations, inhibits lipolysis, and facilitates lipid accumulation (57). It also downregulates the expression of lipoprotein lipase (58). These alterations are likely to contribute to elevated triglyceride and LDL levels while decreasing HDL levels in individuals with vitamin D deficiency (59), representing a significant factor in the association between vitamin D and obesity. The analysis of BMI in this study further revealed a notable negative correlation between vitamin D levels and obesity. The current consensus suggests that there is an interassociation among vitamin D status, inflammation, dyslipidemia, and obesity. Moreover, the association between vitamin D and obesity is regarded as bidirectional (26). On the one hand, as previously discussed, both vitamin D and calcium play critical roles in regulating adipocyte apoptosis, modulating lipogenesis, and enhancing lipid metabolism (60). On the other hand, obesity may lead to diminished serum concentrations of vitamin D. Some studies indicate that for every 10 kg reduction in body weight, the serum 25(OH)D concentration increases by an average of 6 nmol/L (2.4 ng/mL) (61).

Interestingly, regarding uric acid measurements, the optimal group presented slightly lower uric acid concentrations than did the suboptimal group; however, uric acid levels within both the optimal and suboptimal groups did not differ significantly from those observed in the deficient group. The literature supports the notion that serum uric acid is often elevated among patients suffering from diabetes mellitus and metabolic syndrome (62). Importantly, an increase in parathyroid hormone (PTH) due to vitamin D deficiency has been identified as a primary causal mechanism (63, 64). Nonetheless, other influencing factors must also be considered, such as the dietary intake of purines and the renal excretion capacity of uric acid, which may vary according to vitamin D levels; additionally, the influence of vitamin D on uric acid concentrations could be constrained once its level falls below a specific threshold, indicating that the association between vitamin D and uric acid is likely nonlinear. Future research should delve deeper into how urinary uric acid regulation operates under conditions of vitamin D deficiency.

### The association between vitamin D and lifestyle and dietary habits

We observed significant differences in lifestyle and dietary habits between individuals with low vitamin D levels and those with high vitamin D levels. Specifically, the former group reported lower breakfast consumption frequency, shorter sun exposure durations, and a tendency to eat more at night. Additionally, this cohort exhibited increased consumption of sugary beverages, a preference for cold foods, heightened work-related stress, prolonged use of air conditioning during the summer months, and extended daily sedentary periods. These lifestyle factors may collectively influence vitamin D status; notably, the frequency of sugary drink consumption and work pressure emerged as independent risk factors for vitamin D deficiency, whereas daily sun exposure served as an independent protective factor.

With respect to dietary habits, patients with vitamin D deficiency had a lower frequency of breakfast consumption than did those in the optimal group, likely because breakfast is a primary source of vitamin D. Previous studies support our observations by demonstrating an association between vitamin D levels and breakfast habits. A study conducted by Heaher et al. revealed that individuals who skip breakfast at least three times a week exhibit a 1.5-fold increased risk of vitamin D deficiency compared with those with regular breakfast consumption. Milk, meat, and fish are recognized as rich sources of vitamin D, and their intake is positively correlated with the frequency of breakfast consumption (65). Research by Hill KM et al. identified milk, meat, and fish as the primary dietary sources of vitamin D for Americans and Canadians, with ready-to-eat cereals (RTEs) ranking among the top ten sources in the United States. Furthermore, in both countries, vitamin D intake increases with increasing frequency of daily breakfast, which is attributed primarily to increased milk consumption (66). The report from Vatanparast et al. further underscores the pivotal role of dairy products in contributing to vitamin D intake, accounting for 49.1% of daily requirements (67). Additionally, a study by Peter et al., which focused on adolescent diets, indicated that individuals who regularly consume breakfast along with dairy products have significantly elevated average intakes of calcium and vitamin D (68). Together, these clinical studies and statistical findings suggest a positive correlation between habitual breakfast consumption and vitamin D status; this association is largely due to the frequent selection of dairy products such as milk during breakfast, which are often regarded as key sources of vitamin D. In addition, calcium in dairy products may synergistically act with vitamin D to suppress parathyroid hormone (PTH), thereby reducing adipogenesis and the secretion of inflammatory cytokines (69, 70).

In this study, we observed that a greater proportion of individuals with vitamin D deficiency reported the habit of consuming food before bedtime. Although direct research linking bedtime snacking to vitamin D levels is currently lacking, we hypothesize that this dietary behavior may indirectly influence vitamin D status by impacting energy intake and metabolic health. Bedtime eating can lead to excessive caloric consumption, which may adversely affect metabolic markers such as blood glucose and lipid profiles, which are known risk factors for obesity and metabolic syndrome (71, 72). As previously noted, this association could further heighten the risk of vitamin D deficiency. Consequently, nighttime snacking may exert an indirect effect on vitamin D levels through the promotion of metabolic disorders and obesity.

Moreover, the participants with vitamin D deficiency in our study reported a higher frequency of sugary drink consumption. While research examining the association between sugary beverages and serum 25(OH)D concentrations remains limited, existing evidence suggests a negative correlation between these variables. An animal study conducted by Garcia-Contreras et al. revealed that Coca-Cola consumption was associated with reduced serum 25(OH)D concentrations in rats (73). Additionally, Olson’s team demonstrated that high intakes of juice and soda among obese children were correlated with lower serum 25(OH)D levels (74). Furthermore, Duchaine and Diorio revealed an association between high Coca-Cola consumption in premenopausal women and decreased 25(OH)D levels (75). This phenomenon may be linked to the elevated fructose content present in carbonated beverages, which is thought to interfere with vitamin D metabolism (76). Studies have demonstrated that sugary beverages enhance liver uric acid production via fructose metabolism, activate the NLRP3 inflammasome, promote IL-1β release, and concurrently inhibit the activity of vitamin D hydroxylase (CYP27B1), thereby reducing the level of active vitamin D (77). Furthermore, statistical analysis of interaction effects revealed that the anti-inflammatory effect of vitamin D is not uniform across all populations; its ability to reduce SII is stronger in individuals with high sugary beverage consumption. High-sugar diets exacerbate inflammation through metabolic disturbances (e.g., insulin resistance, oxidative stress). In this context, vitamin D may counteract inflammation more effectively through the following mechanisms: (1) Inhibiting the activation of the NF-κB pathway induced by high sugar (the anti-inflammatory effect of vitamin D is more pronounced under conditions of active inflammation) (61). (2) Regulating gut microbiota and metabolites (e.g., short-chain fatty acids) to repair intestinal barrier damage caused by a high-sugar diet (78). As previously discussed, high sugar intake may decrease the bioactivity of vitamin D through fat tissue accumulation or impaired intestinal absorption. However, when vitamin D levels are sufficient, its anti-inflammatory mechanisms may preferentially target high-sugar-induced pathological pathways (e.g., endothelial damage), thus exhibiting more significant protective effects in high-risk populations. These findings suggest that the anti-inflammatory effects of vitamin D are not isolated but interact dynamically with sugary beverage consumption. This “stress-induced enhancement” phenomenon has important implications for combined intervention strategies (e.g., sugar restriction + vitamin D supplementation) in chronic inflammatory diseases such as diabetes and cardiovascular disease.

Moreover, the data from this study indicate that individuals within the deficient group displayed greater tendencies toward consuming cold foods—including uncooked fresh fruits, vegetables, raw meats and frozen items—than did those at optimal levels. Although there is limited research on this association, Klaus Abraham et al.’s findings suggest that despite having lower body fat percentages, raw food enthusiasts exhibit reduced vitamin D3 concentrations, indicating potential nutritional imbalances (79). This observation led us to speculate that preferences for cold foods might indirectly affect vitamin D synthesis through inadequate nutrient intake. There may be complex biological mechanisms underlying the association among cold and raw food, vitamin D, and chronic inflammation. Cold and raw food may harbor a higher load of pathogenic microorganisms, such as Salmonella, which can induce intestinal inflammation and disrupt the composition of the gut microbiota (80). Dysbiosis of the gut microbiota can impair the absorption and metabolism of vitamin D, thereby reducing its levels (81, 82). As previously discussed, vitamin D insufficiency is associated with increased inflammatory responses in the body. Studies have demonstrated that active vitamin D significantly upregulates the expression of the autophagy-related gene Atg16L1, promotes the formation of the autophagy marker protein LC3II, and enhances autophagosome aggregation, strengthening the defense capability of intestinal epithelial cells against Salmonella infection. Moreover, Atg16L1-mediated inhibition of IL-1β expression highlights the dual role of vitamin D in regulating infectious inflammatory responses: it not only activates autophagy to clear pathogens but also suppresses the overproduction of pro-inflammatory cytokines (83). Therefore, cold and raw food may contribute to chronic inflammation by influencing gut microbiota composition and disrupting vitamin D absorption and metabolism.

In terms of lifestyle choices, participants in the vitamin D-deficient category spent more hours utilizing air conditioning during the summer months, although direct studies exploring correlations remain scarce. We hypothesize that societal advancements coupled with evolving lifestyles have led people to spend increased amounts of time indoors under climate-controlled conditions, thereby reducing opportunities for outdoor activities and sunlight exposure. Ultraviolet (UV) rays from sunlight are essential catalysts that promote endogenous vitamin D synthesis. Lagunova et al. reported that summertime UV radiation serves as a key source facilitating vitamin D production, particularly within northern latitudes (84). Adequate sunlight exposure not only elevates immediate concentrations but also aids storage within adipose tissues, providing reserves throughout the winter season (85, 86). The study by Moan et al. further corroborates this perspective, indicating that in northern countries, vitamin D concentrations fluctuate with seasonal variations in UVB radiation (87). Consequently, inadequate sun exposure during the summer months may adversely impact vitamin D status in the subsequent winter season. In regions such as Guangdong, China, persistently high temperatures lead local residents to favour indoor environments equipped with air conditioning, which can result in decreased outdoor activities and limit opportunities for vitamin D synthesis through natural ultraviolet exposure. Thus, we hypothesize that prolonged periods spent in air-conditioned settings may be associated with lower vitamin D levels, particularly during the summer, when this effect is likely to be more pronounced. Additionally, our findings indicate that the daily average duration of sun exposure among individuals deficient in vitamin D was significantly lower than that of the other two groups, further supporting this hypothesis.

The influence of ultraviolet radiation exposure on vitamin D intake was further illustrated by another finding from our study, which revealed that participants with vitamin D deficiency exhibited longer daily sedentary durations. Sedentary behaviors, such as prolonged periods of work or study, diminish outdoor activity and sun exposure, potentially leading to reduced synthesis of vitamin D. Moreover, sedentary behavior is associated with an increased risk of obesity and metabolic disorders; a study conducted by Wu et al. reported a positive correlation between sedentary time and the risk of metabolic syndrome (88). Additionally, research by De Oliveira et al. revealed that obesity may attenuate the efficacy of vitamin D supplementation (26). These findings suggest that sedentary behavior may adversely impact vitamin D levels through decreased sun exposure while exacerbating issues related to obesity and metabolic disorders. In our study, the elevated BMI in the vitamin D deficiency group further supports this association. Research has demonstrated that patients with metabolic syndrome exhibit a 32.7% larger visceral adipose tissue area (VAT) compared to non-metabolic syndrome individuals (89). Moreover, visceral adipose tissue secretes pro-inflammatory cytokines such as IL-6 and TNF-*α*, which activate neutrophils while suppressing lymphocyte function (90, 91). Additionally, the underlying pathology of metabolic syndrome (MetS) involves insulin resistance, which activates the IKKβ/NF-κB signaling pathway, thereby promoting the expression of monocyte chemoattractant protein-1 (MCP-1). This process recruits monocytes to infiltrate adipose tissue, exacerbating inflammation (51). In summary, sedentary behavior may reduce sun exposure, thereby affecting vitamin D levels, and further intensify the adverse interplay among obesity, metabolic inflammation, and vitamin D.

Furthermore, multiple studies have highlighted associations between diminished vitamin D levels and mood disorders. For example, in rheumatoid arthritis patients, a negative correlation exists between anxiety symptom severity and the vitamin D concentration (92). Animal trials lend credence to supporting claims where consistent low-dose supplementation over 14 days yields anxiolytic effects (93). Similarly, clinical evidence has demonstrated that 6 months of supplementation can alleviate anxiety manifestations (94). These findings indicate that vitamin D may exert a beneficial effect on emotional well-being by modulating the pathological processes in brain tissue associated with mood disorders. The association between work stress, vitamin D, and chronic inflammation can be elucidated through intricate neuroendocrine and immune mechanisms. When the body perceives stress, the hypothalamic–pituitary–adrenal (HPA) axis is activated, resulting in elevated cortisol levels (95). Prolonged exposure to high cortisol may suppress the functionality of vitamin D receptors (VDR), while vitamin D deficiency may further exacerbate negative emotions by disrupting neurotransmitter regulation, thereby creating a vicious cycle. Studies have demonstrated that vitamin D supplementation can reduce urinary free cortisol levels and potentially decrease cortisol production by inhibiting 11β-HSD1 enzyme activity, as well as modulating HPA axis function (96, 97). Furthermore, chronic stress activates both the sympathetic nervous system and the HPA axis, leading to the release of norepinephrine and upregulation of pro-inflammatory cytokines such as IL-1, IL-6, and TNF (98, 99). Vitamin D may influence this inflammatory process by regulating intracellular calcium storage, cell signaling pathways, and protecting neurons involved in neurotransmitter secretion, thus establishing a complex biological interplay among work stress, vitamin D levels, and chronic inflammation.

Additionally, this analysis revealed age disparities, with participants classified under deficiencies being younger [55.00 (42.25–66.00)] than their counterparts categorized optimally [62.00 (54.00–68.00)][61.00 (50.50–68.00)] (*p* < 0.05). We postulate that connections that exist correlate with established patterns surrounding diet/lifestyle choices. This could stem largely from excessive pressures confronting today’s youth, paired with unhealthy eating practices and coupled with lengthy sedentary behaviors. A survey assessing white-collar workers’ nutritional statuses conducted in Shanghai noted that adults aged 18 through 44 demonstrated markedly decreased plasma vitamin D concentrations relative to those in children/adolescents/elderly populations (100). We hypothesize that with societal advancement, the duration of individuals’ working hours progressively increases, thereby significantly intensifying work-related stress among young people. Research has demonstrated a substantial correlation between extended working hours and anxiety symptoms (101). Furthermore, as previously noted, there is a negative correlation between vitamin D levels and anxiety symptoms (92).

Moreover, sustained occupational states amplify sitting durations while increasing reliance on artificial cooling systems, concurrently diminishing natural light exposures, which represent principal mediators influencing the reductions observed concerning circulating vitamins. These findings underscore the necessity of tailoring public health strategies addressing diverse demographic segments, emphasizing the importance of fostering healthy balances integrating nutrition/exercise regimens targeting younger cohorts and specifically encouraging outdoor engagements. In conclusion, future inquiries should prioritize investigating implications surrounding youthful demographics, ensuring that they receive the requisite attention given the unique vulnerabilities present therein.

### Strengths and limitations of the study

This study represents a large-scale survey of Chinese adults, providing a comprehensive perspective on the association between vitamin D levels and the systemic immune-inflammation index (SII) within this population. Compared with previous research predominantly focused on Western populations, single-gender cohorts, or elderly individuals, our sample addresses these gaps in the literature. Furthermore, we excluded participants with acute infections, thyroid dysfunctions, severe organ failure, and malignancies—factors that could significantly skew inflammation index results—thereby enhancing the accuracy of our findings. Our study demonstrates methodological innovation by focusing on common dietary and lifestyle factors among modern individuals, including breakfast habits, eating before sleep, preference for cold and raw foods, consumption of sugary beverages, work-related stress, summer air conditioning usage, and sedentary behavior duration. Notably, this is the first study to investigate the correlations between preferences for cold and raw foods, summer air conditioning use, and vitamin D levels. Given the cross-sectional design of this study, while we have analysed the association between vitamin D levels and the SII, as well as the influence of lifestyle and dietary habits on vitamin D levels, we are unable to definitively establish a causal association among these variables. Although we identified several correlations—such as reduced breakfast consumption, shorter sun exposure durations, and increased evening meal frequencies—in individuals with vitamin D deficiency, the causal links between these factors and vitamin D levels remain to be fully elucidated. These behaviors may indirectly impact vitamin D status and inflammation levels by influencing the duration of outdoor activity, nutrient intake, or metabolic health. In addition, the data collection for this study extended over a period of approximately 18 months. Due to constraints in research design and data availability, seasonal adjustments could not be performed. Nevertheless, it is important to highlight that data collection was conducted continuously from August 2020 to January 2022, encompassing a full annual cycle. This long-term sampling approach helps mitigate seasonal fluctuations to some extent. Furthermore, participants in this study were recruited from Guangdong Province, China (approximately 23° north latitude), which lies in the subtropical zone. In this region, ultraviolet B (UVB) radiation remains relatively stable throughout the year, resulting in minimal seasonal variation in vitamin D levels. Additionally, our findings indicate a high proportion of indoor workers among the study population, suggesting reduced exposure to sunlight variations and thus lower susceptibility to seasonal effects on vitamin D synthesis. Moreover, data regarding lifestyle and dietary habits were collected via questionnaires; however, these self-reported measures may introduce subjective bias that further limits our capacity to draw definitive conclusions. Therefore, we recommend that future studies implement more rigorous controls for these variables through methods such as the following:

1. Objective measurement techniques such as wearable devices can be employed to accurately record sunlight exposure.
2. Prospective studies or intervention trials should be conducted to better elucidate causal associations.
3. Conduct repeated measurements across different seasons to more precisely evaluate seasonal variations in vitamin D levels. Integrate month or season covariates into the model, or apply a cosine function to model the solar cycle. Additionally, recruit populations from diverse latitudes (e.g., northern and southern regions) to assess the influence of sunlight exposure duration on the outcomes.
4. The dietary vitamin D intake of the participants was recorded as a covariate to improve the robustness and reliability of the statistical analysis.
5. Detailed food diaries or food frequency questionnaires should be utilized for more precise dietary assessments.

Despite the aforementioned limitations, this study provides valuable insights into the association between vitamin D levels and the systemic immune-inflammation index (SII), while highlighting the critical role of lifestyle and dietary habits in shaping vitamin D status.

## Conclusion

This cross-sectional study of Chinese adults elucidates the associations among serum vitamin D levels, the Systemic Immune Inflammation Index (SII), and lifestyle dietary habits. The primary finding indicates a significant negative correlation between vitamin D levels and the SII, further supporting the potential role of vitamin D in mitigating inflammation risk, particularly among young and middle-aged individuals with serum vitamin D concentrations ≥30 ng/mL. On the basis of the statistical outcomes of this study, we recommend maintaining serum vitamin D levels at 30 ng/mL or above to reduce the risk of systemic inflammation. When vitamin D concentrations are within the range of 20 ng/mL to 30 ng/mL, although the SII values do not significantly differ from those of the other two groups, this finding does not imply that this level of vitamin D is devoid of health benefits. Further research may be necessary to ascertain the impact of vitamin D levels within this range on inflammation and other health outcomes.

Moreover, vitamin D deficiency is significantly negatively correlated with various indicators of diabetes and metabolic syndrome, including hemoglobin A1c (HbA1c), fasting blood glucose, triglycerides, and low-density lipoprotein, while it is positively correlated with high-density lipoprotein cholesterol, especially when the serum vitamin D concentration is ≥30 ng/mL. With respect to lifestyle factors, individuals in the deficient group reported lower breakfast consumption, greater time spent in air-conditioned environments, higher intake of sugary beverages, and reduced sun exposure—all of which are potentially detrimental to their vitamin D status. In summary, the findings from this study not only provide novel evidence for the anti-inflammatory properties of vitamin D but also underscore the critical importance of lifestyle choices in maintaining optimal vitamin D levels. These insights offer valuable guidance for future intervention strategies related to vitamin D. Future research should further investigate the preventive and therapeutic effects of vitamin D supplementation and lifestyle dietary interventions on chronic inflammation, as well as establish the optimal threshold for the association between vitamin D levels and chronic inflammation.

## Data Availability

The raw data supporting the conclusions of this article will be made available by the authors, without undue reservation.
